# A New Electrochemical Method to Determine Tryptophan in Fruit Juices: Development and Validation

**DOI:** 10.3390/foods11142149

**Published:** 2022-07-20

**Authors:** Assefa Takele, José María Palacios-Santander, Miguel Palma

**Affiliations:** 1 Institute of Research on Electron Microscopy and Materials (IMEYMAT), Campus de Excelencia Internacional del Mar (CEIMAR), Department of Analytical Chemistry, Faculty of Sciences, University of Cadiz, Campus Universitario de Puerto Real, Polígono del Río San Pedro S/N, Puerto Real, 11510 Cádiz, Spain; asetak@yahoo.com; 2 Wine and Agrifood Research Institute (IVAGRO), Campus de Excelencia Internacional Agroalimentario (ceiA3), Department of Analytical Chemistry, Faculty of Sciences, University of Cadiz, Campus Universitario de Puerto Real, Polígono del Río San Pedro S/N, Puerto Real, 11510 Cádiz, Spain

**Keywords:** tryptophan, ultra performance liquid chromatography, differential pulse voltammetry, sonogel–carbon electrodes, fruit juice

## Abstract

Tryptophan (Trp) is an essential amino acid usually found in fruit juices. Its determination is necessary for food companies because of its relation to human health. In this work, a new electrochemical method based on sonogel–carbon electrodes (SNGCEs) was developed and validated using an ultra performance liquid chromatography (UPLC) method as a reference method for the determination of Trp in fruit juices. Cyclic voltammetry (CV), chronoamperometry, and differential pulse voltammetry (DPV) techniques were applied to investigate the oxidation of Trp on a previously polarized SNGCE surface in a Britton–Robinson (BR) buffer solution at pH 3.6. The operating conditions for electroanalysis were optimized using a Box–Behnken design (BBD), obtaining an oxidation peak for Trp at 0.749 V. The linear range for this method was from 0.1 to 5 mg/L. The intraday and interday precision, expressed as a relative standard deviation (RSD), were 3.1% and 2.7%, respectively. The average recovery was 99.01%, and the limit of detection and quantitation were 0.33 and 1.09 mg/L, respectively. Therefore, from the quality analytical parameters obtained, it can be concluded that the new electrochemical method can be successfully used for the routine analysis of Trp in fruit juices. As far as we are concerned, this is the first time that a methodology for Trp determination was performed in this kind of real food matrices.

## 1. Introduction

Tryptophan (Trp) is an essential amino acid [1,2] with various physiological roles: it can function independently or by incorporation into the structure of larger molecules or polymers, such as proteins [1,3]. Trp is required for the biosynthesis of proteins, and it is important in nitrogen balance and the maintenance of muscle mass and body weight in humans [4]. It is also a precursor of many biologically active substances, such as serotonin, 5-hydroxytryptamine (5-HT, a monoamine neurotransmitter), melatonin (a neurohormone), vitamin B3 (niacin) [4,5], and kynurenine (a pathway in which 95% of Trp is catabolized) [6]. If there is a deficiency of vitamin B6 in the body, Trp may be transformed into vitamin B3. The production of vitamin B3 is very important since the body considers its production to be more important than that of serotonin. Therefore, if the body is not getting enough vitamin B3 from the diet, it uses up its available Trp, which will lead to a deficiency of serotonin and melatonin [5]. An abnormal level of serotonin can cause depression, while melatonin is associated with sleeping disorders and Alzheimer’s and Parkinson’s diseases [7]. Therefore, the daily intake of Trp is an important matter for human health.

As mentioned before, Trp is an essential amino acid, so it is commonly added as a food fortifier in dietary food products and in pharmaceutical products because it is scarcely present in vegetables and cannot be directly synthesized in the human body [1,4,8]. Trp is essential for humans and acts predominantly as the precursor of serotonin, which is an important neurotransmitter with physiological activity [3,9] for the transduction of a neurochemical signal between neurons. The synthesis of serotonin in the brain is dependent on the availability of its precursor, the Trp amino acid. Serotonin is considered essential in the modulation of several behavioral and physiological functions, such as mood, sleep and wakefulness, sexual behavior, cognition, appetite, impulsivity, aggression, neurodevelopment, circadian rhythms, body temperature, and neuroendocrine function [10]. Therefore, Trp plays also an important role in physiological processes, such as nerve transmission and immune response, and pathological processes, such as stress, depression, sleep, and appetite disorders, because of its relationship with serotonin [3]. However, improper metabolism of Trp results in toxic products that can be accumulated in the brain, and which can then cause problems, such as hallucinations, delusions, and schizophrenia. If Trp is taken in high doses, it may show side effects, such as agitation, confusion, diarrhea, fever, and nausea [1].

In the past, various methods were developed for the determination of Trp in food samples, such as spectroscopy, high performance liquid chromatography (HPLC), fluorimetric methods, capillary electrophoresis and electroanalysis [11], flow injection chemiluminescence [9,12], and colorimetric methods [13]. Some of the previously reported methods for the determination of Trp in different samples are shown in Table S1.

The electrochemical detection method was developed as a potentially useful technique for pharmaceutical applications [1]. It has been found to be a more attractive technique for the determination of electroactive compounds in biological samples and foodstuffs because of its sensitivity, fast operation, reproducibility, accuracy, low cost, very small sample, and solvent consumption. In fact, modern electrochemical methods are more regularly used for the study of industrial and environmental applications and drug analysis in dosage forms and biological samples [14].

As shown above in Table S1 and other reported methods, Trp was determined in different food samples, different kinds of biological samples, and pharmaceutical preparations using various analytical methods. However, there few applications related to the determination of Trp in fruit juices that have been reported.

Fruit juices play an important role worldwide in the human diet, and the industry has become large and profitable [15]. When fruit juices are consumed in moderation as part of a balanced diet, they have a positive effect on promoting health and reducing disease risk [16]. As fruit juices are healthier choices among consumers, the quality and the safety of juice products are always a worry, and they are always subjected to very detailed legislation, ensuring all the necessary information on their nutritional benefits and consumptions [17]. Therefore, the development of a simple, sensitive, and less expensive detection method for Trp is of great significance and interesting to public health and food companies. Thus, the aim of this study was to develop an electrochemical method using sonogel–carbon electrodes for the direct determination of Trp in different fruit juices (apple; pineapple; tropical; five fruits; mixture of pineapple and grape; mixture of peach and grape; mixture of pineapple, apple, and grape; and mixture of peach, apple, and grape) and to validate the methodology using ultra performance liquid chromatography (UPLC) as a reference method. To the extent of our knowledge, this is the first time that Trp determination has been accomplished in fruit juices using this analytical technique.

## 2. Materials and Methods

### 2.1. Chemicals and Reagents

Tryptophan standard (T0254-5G, reagent grade, purity ≥ 98%) was obtained from Sigma-Aldrich (Sigma-Aldrich Co., St. Louis, MO, USA). HPLC-grade methanol, acetonitrile, acetic acid, trimethoxymethylsilane, hydrochloric acid, sulfuric acid, phosphoric acid, and boric acid were purchased from Merck KGaA (Darmstadt, Germany); sodium hydroxide from Panreac Quimica SAU (Barcelona, Spain); graphite powder from Alfa Aesar (Kandel, Germany); and gold (III) chloride trihydrate (HAuCl_4_) from Sigma Aldrich (St. Louis, MO, USA). All the reagents were of analytical grade and used without further purification. Purified water was obtained from the Milli-Q System (Billerica, MA, USA). Samples of fruit juices were purchased from local supermarkets (Mercadona and Supersol) in Puerto Real, Cadiz, Spain.

### 2.2. Preparation of Buffer, Tryptophan Standard Solution, and Real Samples

An amount of 0.04 M Britton–Robinson (BR) buffer solution was used as an electrolyte and placed in the electrochemical cell. The pH of the solution was adjusted using a pH meter (Crison GLP21). The buffer solution at pH 3.6 was prepared from a mixture of 2.3 mL of acetic acid (CH_3_COOH), 2.7 mL of phosphoric acid (H_3_PO_4_), and 2.48 g of boric acid (H_3_BO_4_) in a 1 L volumetric flask and by diluting to a volume with Milli-Q water. Then the pH of the buffer solution was adjusted to 3.6 using sodium hydroxide (NaOH). The prepared BR buffer solution was used as a blank in the determination of Trp. The pH of the buffer solution was adjusted to 3.6, considering the pH’s of the fruit juices to be determined, which were in the range of 3.3 to 3.8.

A Trp standard stock solution (100 mg/L) was prepared in methanol. Working standard solutions were prepared from stock solution at concentration levels ranging from 0.1 to 5 mg/L in a 50:50 (*v*/*v*) Milli-Q water/methanol mixture.

Different types of fruit juice samples were purchased from local supermarkets, Mercadona (apple, pineapple, tropical, five fruits, mixture of pineapple and grape, and mixture of peach and grape), and Supersol (apple; mixture of pineapple, apple, and grape; and mixture of peach, apple, and grape). Apple; pineapple; mixture of pineapple and grape; and mixture of pineapple, apple, and grape were prepared directly by filtering through a 0.45 µm filter, then filtered through a 0.22 µm filter, transferred to a vial, and injected into the UPLC system. Other fruit juice samples—tropical; five fruits; peach; and mixture of peach, apple, and grape—were first clarified by centrifugation (4000× *g*, 5 min), filtered through a 0.45 µm filter and then through a 0.22 µm filter, transferred to a vial, and injected to the UPLC system. As it is not required by law, no information about the levels of Trp appeared in the commercial labels.

### 2.3. Preparation of Sonogel–Carbon Electrodes

Sonogel–carbon was synthesized by mixing 500 µL of trimethoxymethylsilane and 100 µL of HCl 0.2 M, as reported in [18]. The mixture was homogenized using a high-power ultrasound generator, Sonicator 3000, from Misonix (Misonix, Inc., Farmingdale, NY, USA) (equipped with a 13 mm titanium tip), which provides a maximum power of 600 W. In this case, the energy applied was between 90 and 100 joules. After sonication, 0.5 g of graphite powder was added to the mixture and well homogenized. Then the SNGCEs were prepared by inserting the material obtained into glass capillary tubes (internal diameter of 1.15 and external diameter of 1.55 mm) and connecting the ceramic material to a copper wire for electric contact.

### 2.4. Polarization and Modification of Sonogel–Carbon Electrodes

The measurements of standard solutions and quantitation of tryptophan in fruit juice samples were carried out using a SNGCE. Before conducting the measurements, SNGCEs were well polished with a waterproof silicon carbide paper (FEPA P#1200, Struers, Germany) and white paper until the surface become shiny. Then the polished electrodes were polarized amperometrically in 25 mL of 0.1 M H_2_SO_4_ in an electrochemical cell. The polarization was performed as follows: The working electrode potential was poised at −0.7 V for 10 s, and then stepped at +1.8 V for 10 s, the procedure being repeated 8 times, as reported previously [19,20]. After that, the SNGCEs were conditioned in a 4 mL blank solution from −0.5 to 1.2 V, with a step potential of 0.005 V, at a scan rate of 0.05 V/s, using cyclic voltammetry technique, being then ready for measurements.

The SNGCEs were modified using two approaches: electrodeposition from a gold solution (0.05 mM of HAuCl_4_) and drop casting of gold nanoparticles (AuNPs, 4 µL of AuNP colloidal solution) onto the surface of SNGCE [21]. A gold solution (0.05 mM of HAuCl_4_) was prepared by dissolving 0.005 g HAuCl_4_ in a H_2_SO_4_ 0.5 M solution and diluting to a volume with the same solution in a 25 mL volumetric flask. Then the electrodeposition was performed by polarizing the SNGCE in 25 mL of the previous solution using amperometry technique. Additionally, the effect of duration time on the electrodeposition of a gold solution on the electrode surface was studied by setting the duration time at 200, 600, and 1000 s under the amperometric conditions.

### 2.5. Chromatographic Conditions

An ACQUITY UPLC^®^ H-Class system coupled to an Acquity UPLC^®^ Fluorescence Detector and the system was controlled by Empower^TM^ 3 Chromatography Data Software (Waters Corporation, Milford, MA, USA). The Acquity UPLC system was equipped with a binary solvent manager, a sample manager including the column heater, an optional sample manager, pumps, and the detector.

Analysis of tryptophan standard solution and real samples (fruit juice samples) was performed using an Acquity UPLC^®^ H-Class system coupled to an Acquity UPLC^®^ Fluorescence Detector, and the system was controlled by Empower^TM^ 3 Chromatography Data Software (Waters Corporation, Milford, MA, USA). Separations were performed using a reverse-phase RP C18 Cortecs UPLC^®^ Solid-Core-Based (SCB) Column (silica-based solid-core particle, 100 mm length, 2.1 mm ID, 1.6 µm particle size) obtained from Waters. Identification of Trp was carried out by setting the photodiode array detector in the wavelength range of 210–400 nm for the 3D scan and set at 278 nm for peak integration. Quantitation of real samples was carried out, setting the fluorescence detector (FD) at an excitation wavelength, λ_ex_ = 280 nm, and an emission wavelength, λ_em_ = 325 nm. The FD sensitivity for the 3D scan was set at PMT gain 1000. The mobile phase was a binary solvent system consisting of phase A (water with 2% acetic acid) and phase B (acetonitrile with 2% acetic acid). The injection volume of the sample was 1.5 µL, and the flow rate was 0.6 mL/min. The analyses were performed at 47 °C (column temperature) for 4 min using a gradient elution (Table 1). The column was equilibrated for 3 min before starting injections. The samples were filtered before injections using a 0.45 µm filter and then filtered through 0.22 μm syringe-driven filter. Under these conditions, the resulting retention time for Trp was 2.64 min for 11 concentration levels ranging from 0.1 to 5 mg/L.

### 2.6. Electrochemical Measurements

Differential pulse voltammetry (DPV) and cyclic voltammetry (CV) experiments were carried out using an Autolab PGSTAT128N (Ecochemie, Utrecht, The Netherlands) potentiostat/galvanostat connected to 663 VA stand (Metrohm, Swiss) and a personal computer. The GPES program version 4.9 software was used to generate signals, data analysis, and storage. A three-electrode electrochemical system composed of a platinum wire as the auxiliary (counter) electrode, Ag/AgCl (KCl 3 M) electrode as the reference electrode, and unmodified sonogel–carbon electrode (SNGCE) as the working electrode was employed.

The calibration curve for Trp standard solution and the quantitation of real samples were performed using differential pulse voltammetry. The DPV measurements were performed in Britton–Robinson buffer by setting the interval time at 0.6 V, step potential at 0.016 V, and modulation amplitude at 0.1 V; the solutions were magnetically stirred for 20 s.

### 2.7. Electrochemical Analysis of Tryptophan

The calibration curve for Trp standard solution and the quantitation of real samples were performed using differential pulse voltammetry. After the polishing, amperometrically polarizing, and CV conditioning of the SNGCE, the DPV measurements were performed by adding Trp standard solution into an electrochemical cell containing 4 mL of Britton–Robinson buffer and stirring the solution for 20 s with a magnetic stirrer. Real samples were determined, first, running the blank. Subsequently, the blank was discarded, and 4 mL of a real sample was added into the electrochemical cell and stirred for 20 s with a magnetic stirrer. After each measurement of the real sample, the SNGCE was repolished, repolarized, and reconditioned. This methodology ensured the reproducibility and repeatability of the measurements, avoiding fouling of the electrode surface.

### 2.8. Experimental Design and Statistical Analysis

A Box-Behnken design (BBD) is a kind of experimental design commonly used to optimize successfully experimental variables [22,23]. In this work, it was employed to measure the effect of three independent variables: modulation amplitude (×1), step potential (×2), and interval time (×3) on the determination of Trp using DPV. It is supposed that the independent variables are continuous and controllable during the experiments. Three levels were selected for each factor, being coded as −1, 0, and +1 (low, central, and high value), respectively. The values considered for each variable were: modulation amplitude at 0.01 (−1), 0.055 (0), and 0.1 (+1) V; step potential at 0.004 (−1), 0.010 (0), and 0.016 (+1) V; and interval time at 0.2 (−1), 0.4 (0), and 0.6 (+1) s.

The matrix experiment based on BBD was established by combining the treatment variables at the midpoints of the edges of the process space and the center, thus avoiding extreme treatment combinations, which are the advantage of the design. The number of experiments required was calculated by applying the particular BBD equation [24]: N = 2k (k − 1) + C_0_, where N is the total number of experiments required, k is the number of factors (×1, ×2, ×3), and C_0_ is the number of central points. Accordingly, N = 2 × 3 (3 − 1) + 3 = 15, the design consisting of 15 experimental points, including 3 central points, was used to assess the effects of three variables and the interaction effects between variables on responses by fitting the data to a polynomial model [24,25,26].

A Trp standard solution of 0.5 mg/L was measured by performing 15 experimental runs. Statgraphics^®^ Centurion software was used to design the experiment and to calculate the model from the experimental data that best describes the variation of the signals. For this purpose, analysis of variance (ANOVA) was performed at a significance level of 95% (*p*-value = 0.05). A *t*-value of 2.571 was established to evaluate which independent variable was statistically significant.

### 2.9. Method Validation

A variety of general validation protocols have been recommended by organizations, such as the Food and Drug Administration (FDA) and the International Conference on Harmonization (ICH). Consequently, the validation of the developed electrochemical method and the proposed UPLC method for the analysis of Trp was carried out as outlined by FDA [27] and ICH [28,29] guidelines. The validation parameters used were linearity, precision, accuracy, limit of detection, and limit of quantitation.

**Linearity and range**

The linearity of Trp was determined by addition method. A working solution at 11 concentration levels ranging from 0.1 to 5 mg/L was added from a standard stock solution of Trp (100 mg/L) into the electrochemical cell containing 4 mL of the blank (BR buffer solution) for DPV measurements.

**Precision**

Repeatability, as recommended by the ICH guideline, is determined from a minimum of 9 determinations covering the specified range of the procedure (e.g., 3 concentrations, 3 replicates each) or from a minimum of 6 determinations at 100% of the test or target concentration [28,29].

The precision of the proposed method was determined as repeatability (intraday precision) and intermediate precision (interday) and expressed in terms of percentage relative standard deviation (%RSD) of the peak current. Repeatability was determined from 10 replicate measurements (*n* = 10) of Trp standard solution (2.5 mg/L), and intermediate precision was analyzed at different concentration levels in triplicate (*n* = 3) on 3 separate days.

**Accuracy and recovery**

The accuracy of the method was determined by standard addition. It was determined by spiking the real sample with standard Trp solution. Then the recovery was calculated as %RSD from the peak current. 

**Limit of detection and limit of quantitation**

Limit of detection (LOD) and limit of quantitation (LOQ) for Trp were calculated from the equation of regression analysis obtained from the calibration curve as follows: LOD = 3σ/S and LOQ = 10σ/S, where σ is the standard deviation of the intercept and S is the sensitivity expressed by the slope of the calibration curve [30,31].

## 3. Results

### 3.1. Optimization of the Electrochemical Method

#### 3.1.1. Modification of Sonogel–Carbon Electrodes

As it is well known, the modification of the electrode surface with some kind of nanomaterials, for instance, gold nanoparticles, affects positively the analytical signal [32]. Therefore, different procedures were evaluated for enhancing the Trp signal and sensitivity of SNGCEs. This includes electrodeposition of a gold solution and drop casting of AuNPs onto the surface of the electrodes. Then the modified electrodes and bare electrodes were used to determine the Trp standard solution at concentration levels of 0.1 to 10 mg/L. Calibration curves were constructed from DPV measurements, and the sensitivities of modified and unmodified electrodes are given in Table 2. Duration in the table means the time required for the modification of the electrode using the electroanalytical technique (chronoamperometry). After comparing the results, bare (unmodified) electrodes showed better sensitivity and signal than the modified electrodes. The sensitivity of the bare electrodes is almost 75% higher than that of the modified electrodes. The standard deviation (SD) in this case is referred to as a measure of repeatability of the modified or not modified sensor. The bare electrode is much more sensitive by far, and the slight increase in SD with respect to Au-deposited electrodes is not a demerit in the future electroanalytical performance of this bare electrode, when compared with the deposited ones. Here, the deposition with gold does not offer improvement in Trp detection. Thus, the determination of Trp was carried out using an unmodified SNGCE.

**Figure 1:**
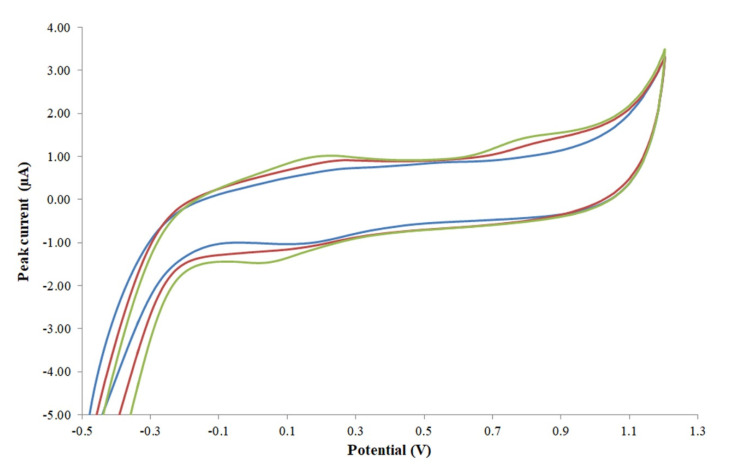
Voltammograms of different SNGCEs obtained from CV scanning: ‘blue: after polishing and polarizing 1’, ‘red: after polishing and polarizing 2’, ‘green: after polishing and polarizing 3’.

It was also observed that the reproducibility of the electrode was improved when the electrode was repolished and repolarized after each measurement and having a comparable voltammogram (Figure 1) from the CV scanning.

**Figure 2:**
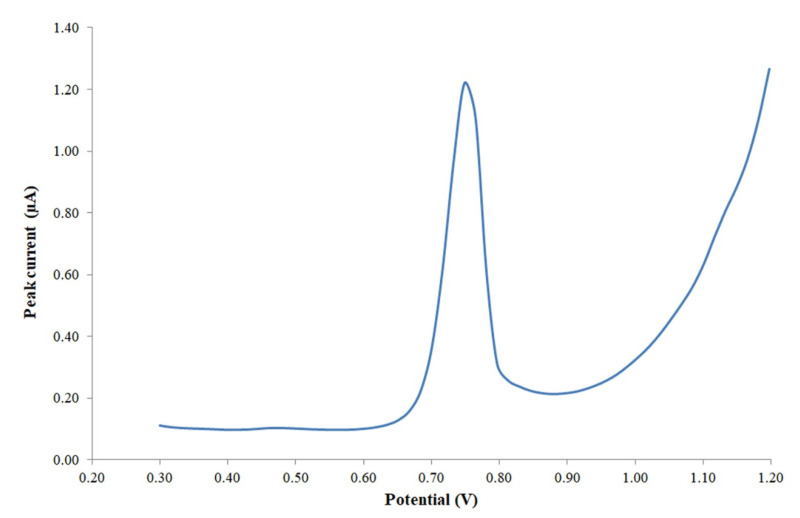
Voltammogram with the final peak signal of tryptophan (2 mg/L) after adjusting the measurement conditions.

In order to avoid the early oxidation of other species in the medium at potential values lower than 0.3, which could affect the Trp signal, and to obtain a good shape for the peak signal, the initial potential was set at 0.3 V and the end potential at 1.2 V for all the electroanalytical measurements. After that, a peak current corresponding to Trp was observed at a potential of about 0.749 V, and a typical voltammogram obtained during the calibration analysis is shown in Figure 2.

#### 3.1.2. Optimization of DPV Conditions Using the Box–Behnken Design

**Figure 3:**
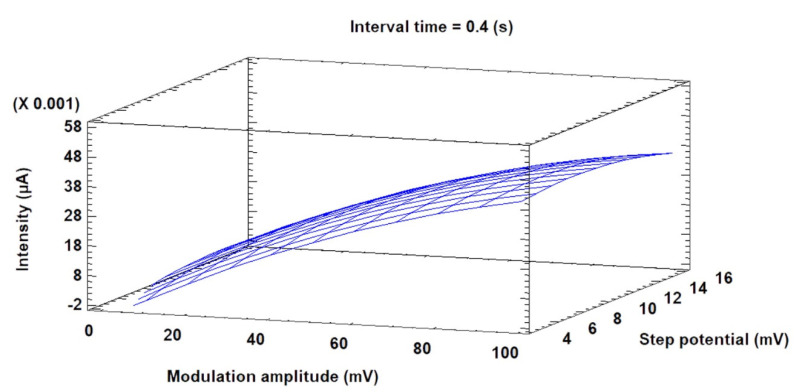
Surface plot of current response.

The differential pulse voltammetric conditions were optimized using the BBD. The optimization process was carried out according to a previous work [32] in order to maximize the current intensity (used as the response variable) (see Section 2.9 for more details). Hence, after performing 15 experimental runs (Table 3), the data obtained were used to build a model (adjustment of 90%), where the optimal values extracted for the variables were as follows: modulation amplitude = 0.1 V, step potential = 0.016 V, and interval time = 0.6 s. Under these conditions, improved signals (with optimized current intensities or peak currents) were obtained. The surface plot obtained from the experiments is shown in Figure 3. The response surface indicates that the anodic current intensity is maximized at the highest modulation amplitude and step potential values using intermediate values of the interval time variable.

### 3.2. Validation of the Electrochemical Method

Linearity, LOD, LOQ, and intraday and interday precision were used to validate the new method. A Trp standard solution was analyzed at 11 concentration levels in the range of 0.1 to 5 mg/L, and a good linear regression was obtained between peak current and concentration. The correlation coefficient, y-intercept, and slope of the regression line were then determined. It was observed that using the optimized conditions, the slope (i.e., the sensitivity) was increased from the starting measures (Table 2). The analytical characteristics for the different concentration ranges are given in Table 4.

LOD and LOQ were determined from the calibration data using regression analysis and were found to be 0.33 and 1.09 mg/L, respectively. The sensitivity of the electrode expressed as the slope of the calibration curve (0.63 µA·L/mg) was also improved as compared with the sensitivity (0.43 µA·L/mg) shown in Table 2.

The values of RSD were calculated to determine intraday and interday precision. The results showed that the RSD values for intraday and interday precision of the Trp standard solution were found to be less than 3.15% and 2.75%, respectively. The RSD value for the intraday precision of a real sample was 4.06% (Table 5). Therefore, the repeatability of the detection of the Trp concentration on the electrode was excellent.

The accuracy for Trp was determined by standard addition method as percent recovery. Recovery values (%) were calculated from the peak current, and the average recovery (*n* = 3) was found to be 99.01%, which indicates that excellent recovery was obtained. Thus, the developed method may be successfully used for the determination of Trp in fruit juices with adequate accuracy.

It is also very important to remark that after consecutive measurements, the signal of Trp decreases due to fouling of the analyte on the electrode surface. Previous studies [33] reported that Trp, like most other organic molecules, can be easily adsorbed onto electrodes and can foul the electrode during successive scans. The fouling effect of bare glassy carbon electrodes (GCEs) was higher than that of modified electrodes. There was a decrease of almost 30% of the initial response after the 10th consecutive cycle in a tryptophan standard solution using the modified electrode, whereas the decrease of the same degree was observed after only the 3rd cycle using bare GCEs. This demonstrates that the fouling effect was much higher in bare GCEs than in modified electrode. In our case, it seems that this situation can be also corroborated (see Section 3.4). The redox mechanism involving Trp oxidation at a SNGCE, as concluded after CV results varying the scan rate, indicates that Trp might need not only to diffuse from a solution to the electrode surface, but also to interact with the surface before suffering the oxidation process, and that this interaction could control the rate of the redox process. This fact makes too much probable the existence of a fouling effect during Trp determination at a sonogel–carbon electrode. Therefore, adequate methodology, as the one used here, based on polishing, polarization, and CV conditioning of SNGCEs, was necessary before determining the analyte with our sensing system.

### 3.3. Study of the Electrochemical Reaction Mechanism

**Figure 4:**
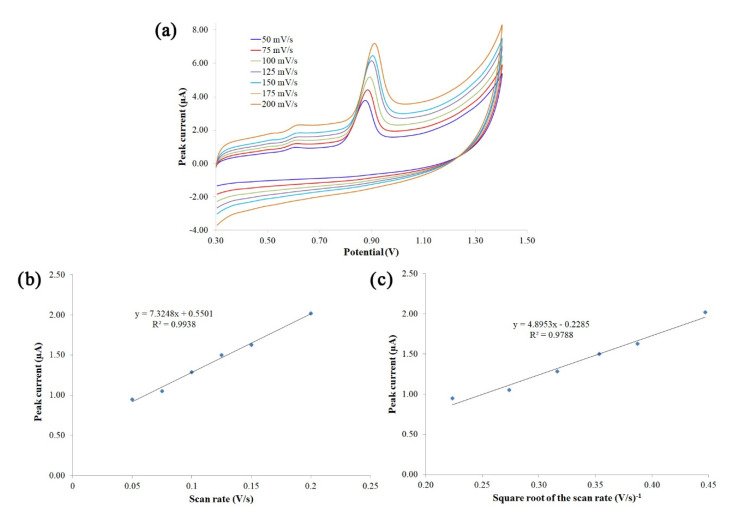
Cyclic voltammograms of tryptophan at different scan rates (**a**) (50 (blue), 75 (red), 100 (light green), 125 (purple), 150 (light blue), and 200 (orange) mV s^−1^), plot of peak current vs. scan rate (**b**), and plot of peak current vs. square root of scan rate (**c**).

The results obtained in this section support the new method proposed to determine Trp. To understand the electrochemical reaction mechanism of Trp, the effect of scan rate on the oxidation peak currents of Trp at an unmodified SNGCE was investigated. The electrode was well polished and polarized in 25 mL of 0.1 M H_2_SO_4_ using amperometric conditions. Then, the CV measurement for a Trp standard solution of 2 mg/L in a BR buffer solution (pH 3.6) was performed at different scan rates of 50, 75, 100, 125, 150, and 200 mV s^−1^. Subsequently, the signals were recorded and the oxidation peak current measured (see Figure 4a). It was observed that the oxidation peak potential of Trp shifts positively with the increase in scan rate, as reported in the literature [34,35]. These shifts may be attributed to the accumulation of the oxidation or reduction products on the electrode surface [35]. As shown in Figure 4b, a linear relationship between the oxidation peak current (I_p_) and the scan rate (*v*) was obtained: *I_p_* = 7.3248*v* + 0.5501 (*I_p_*: µA, *v*: V s^−1^), with a correlation coefficient of R = 0.9938, in the range of 50–200 mV s^−1^ and in a BR buffer solution (pH 3.6). This fact indicates that the electrochemical oxidation of Trp at the SNGCE was an adsorption-controlled process [1,34,36,37]. Similarly, a linear relationship was obtained between the oxidation peak current and the square root of the scan rate: *I_p_* = 4.8953*v* − 0.2285 (*I_p_*: µA, *v*: V s^−1^), with a correlation coefficient of R = 0.9788 (Figure 4c), suggesting that the electrochemical oxidation of Trp was a diffusion-controlled process. Besides, the plot of logarithm of the peak current versus the logarithm of the scan rate gave a straight line with a slope close to a theoretical value of 0.6 (log *I_p_* = 0.5738 log *v* − 5.3088; R^2^ = 0.9816), which indicates a reaction with a mechanism controlled by a mixed adsorption/diffusion electrode process [35]. According to the results obtained and due to the fouling suffered by the electrode surface, it can be concluded that the electrochemical process of Trp consists of a mixture of both adsorption and diffusion-controlled processes. These results serve to corroborate the responses obtained and the electroanalytical methodology proposed for Trp determination.

### 3.4. UPLC Determination of Tryptophan in Fruit Juice Samples

Based on the previously developed and validated UPLC method [38] with a fluorescence detector, Trp was quantified in different fruit juice samples. The retention time of Trp was found to be about 2.64 min (see Figures S1 and S2 in the Supplementary Materials section).

The UPLC method was validated for linearity, precision, accuracy, LOD, and LOQ according to the ICH and FDA guidelines.

The linearity of the method was determined by analyzing a series of standard solutions of tryptophan following the same conditions stated in Section 2.9. The signals obtained were proportional to their concentrations within these ranges. The equation for the calibration curve was y = 411,296 × −258,786 with a linear correlation coefficient (r) of 0.9996.

LOD and LOQ for Trp were calculated from the equation of regression analysis obtained from the calibration curve based on the standard deviation of the response and the slope of the calibration curve. The limit of detection and limit of quantitation were found to be 0.09 and 0.29 mg/L respectively.

The precision of the UPLC method was determined as repeatability (intraday precision) from 10 independent analyses (*n* = 10) of the fruit juice samples on the same day. Repeatability was also determined from 5 independent analyses (*n* = 5) by running the blank between each sample. The percentage relative standard deviations were 1.77% and 1.01%, respectively.

Moreover, the effect of the filtration of fruit juices on the recovery was assessed by spiking a standard solution of 1 mL (5 mg/L) with 5 mL of a pineapple juice sample before filtration and after filtration in triplicate (*n* = 3). It was observed that good recoveries were obtained in both cases. The spiking of the standard solution before the filtration of the real sample and then the filtering through 0.45 and 0.22 µL filters gave a recovery of 117%. Meanwhile, the spiking of the standard solution after the filtration of the real sample with 0.45 µL and then the filtering of the spiked sample through 0.22 µL gave a recovery of 115%. This ensured that the filtration of the fruit juice samples before spiking or after spiking has no significant effect on the analysis results. Thus, the method can be successfully used for the determination of Trp in fruit juice with adequate accuracy.

### 3.5. Comparison of UPLC and Electrochemical Methods

The developed electrochemical method was compared with the reference UPLC method with respect to method validation parameters (Table 6), and the results showed that both methods are reproducible and can be successfully used in a food quality control laboratory. As shown in the table below, UPLC has lower LOD and LOQ and, similarly, better recovery and intraday precision as compared with the electrochemical method. However, the electrochemical method is faster and requires less expensive systems.

The developed electrochemical method was used for quantifying tryptophan in fruit juice samples, as an additional step in the validation process. The extent of our knowledge, this is the first time that tryptophan has been determined electrochemically in fruit juices. Results from the UPLC method were used as a reference method. Tryptophan was quantified in fruit juices that were packed in a container containing 200 mL, and the results are shown in Table 7. A fruit juice containing a mixture of pineapple and grape showed the maximum concentration, and an apple juice sample showed the minimum concentration in both the reference method (UPLC) and the developed electrochemical method. It must be noted that for those samples containing apple juice, either pure or mixed with other juices, the electrochemical method produced higher results than the chromatographic method. It can be concluded that the samples elaborated with apple juice contained some kind of interference that affected the electrochemical signal. Those interferences were separated using the chromatographic method.

## 4. Conclusions

An electrochemical method using a sonogel–carbon electrode was developed, optimized, and validated for the determination of Trp in fruit juices using ultra performance liquid chromatography as a reference method. 

Sonogel–carbon electrodes were prepared as an electrochemical sensor for the determination of Trp. The developed electrochemical method was sensitive, rapid, cheap, and easy to use, and this is the first time that Trp determination was accomplished in fruit juices using the proposed methodology. Moreover, this methodology avoids fouling effects due to the Trp nature with an adequate combination of mechanical and electrochemical cleaning procedures of the electrode surface; that is why electrode surface modification is not recommended. The sample preparation needs only a previous filtration step as in UPLC; no other pretreatment is performed on the juice sample. The proposed method has shown many desirable properties for the determination of Trp, including good limit of detection and linear range, good reproducibility and repeatability, good recovery, and good sensitivity. The LOD and LOQ were 0.33 and 1.09 mg/L, respectively, for the developed electrochemical method. Additionally, the recovery was 99.01%, and the RSDs for intraday and interday precisions were 3.12% and lower than 2.75%, respectively. Comparison with the reference method demonstrated good agreement for the results of Trp for those fruit juices without apple juice; therefore, it can be successfully used in food quality control for most fruit juices.

## Figures and Tables

**Table 1:** Elution gradient for tryptophan determination by UPLC (phase A: water with 2% acetic acid; phase B: acetonitrile with 2% acetic acid).

	Time (min)	Flow Rate (mL/min)	Phase A (%)	Phase B (%)	Curve
1	Initial	0.6	100.0	0.0	Initial
2	1.00	0.6	100.0	0.0	6
3	3.00	0.6	95.0	5.0	6
4	4.00	0.6	90.0	10.0	6

**Table 2:** Sensitivity of modified and unmodified SNGCE.

SNGCE	Duration (s)	Sensitivity (*n* = 2) (µA·L/mg)	SD
Gold solution electrodeposited (HAuCl_4_)	200	0.18	0.009
	600	0.11	0.006
	1000	0.14	0.012
Gold nanoparticles drop-casted (AuNPs)		0.13	0.005
Bare (unmodified)		0.43	0.042

SNGCE: Sonogel–carbon electrode; SD: standard deviation.

**Table 3:** Box–Behnken design for optimizing electroanalytical experimental conditions.

Experiments	Modulation Amplitude (MA, mV)	Step Potential (ST, mV)	Interval Time (IT, s)	Coding Variables (MA, ST, and IT)			Current Intensity (μA)
1	100	16	0.4	+1	+1	0	3.50 × 10^−2^
2	10	10	0.2	−1	0	−1	6.03 × 10^−3^
3	100	10	0.6	+1	0	+1	4.66 × 10^−2^
4	55	16	0.2	0	+1	−1	1.42 × 10^−2^
5	55	4	0.2	0	−1	−1	1.12 × 10^−2^
6	55	10	0.4	0	0	0	2.84 × 10^−2^
7	10	4	0.4	−1	−1	0	4.53 × 10^−3^
8	10	16	0.4	−1	+1	0	5.98 × 10^−3^
9	55	10	0.4	0	0	0	3.13 × 10^−2^
10	55	4	0.6	0	−1	+1	3.17 × 10^−2^
11	55	16	0.6	0	+1	+1	3.84 × 10^−2^
12	100	4	0.4	+1	−1	0	3.52 × 10^−2^
13	55	10	0.4	0	0	0	2.76 × 10^−2^
14	100	10	0.2	+1	0	−1	1.37 × 10^−2^
15	10	10	0.6	−1	0	+1	4.81 × 10^−3^

**Table 4:** Analytical characteristics for the determination of tryptophan.

Linearity Range (mg/L)	0.1–5
Regression equation	Y = 0.6273 × −0.2523
Determination coefficient (r^2^)	0.9880
Correlation coefficient (r)	0.9940
Intercept (b) (µA)	0.2523
LOD (mg/L)	0.33
LOQ (mg/L)	1.09

**Table 5:** Intra- and interday precision for tryptophan (SD: standard deviation; RSD: relative standard deviation).

Intraday Precision (*n* = 10)			Interday Precision (*n* = 3)	
Concentration (mg/L)	SD	RSD (%)	SD	RSD (%)
2			1.19 × 10^−8^	1.21
2.5	3.94 × 10^−8^	3.12	3.19 × 10^−8^	2.51
3			4.54 × 10^−8^	2.73
Real sample (*n* = 7)	3.06 × 10^−8^	4.06		

**Table 6:** Method validation parameters for the determination of tryptophan in fruit juices.

Parameter	UPLC	Electrochemical
Linear range concentration (mg/L)	0.09–5	1.09–5
Coefficient of determination (r^2^)	0.999	0.988
Intraday precision (*n* = 9), RSD (%), real sample	1.77	4.06
Intraday precision (*n* = 10), RSD (%), standard		3.12
Recovery (%)	116.18	99.01
LOD (mg/L)	0.09	0.33
LOQ (mg/L)	0.29	1.09

**Table 7:** Concentration of tryptophan in some analyzed fruit juices.

Real Samples	Electrochemical	UPLC
	Conc. (mg/L)	Conc. (mg/L)
Pineapple	2.56	1.84
Peach + grape	1.31	1.15
Apple	1.34	0.45
Peach + apple + grape	1.22	0.65
Peach	1.13	1.21

## Data Availability

Data is contained within the article or supplementary material.

## Supplementary Materials

The following supporting information can be downloaded at: https://www.mdpi.com/article/10.3390/foods11142149/s1: Figure S1: Chromatogram of tryptophan obtained during the calibration curve; Figure S2: Chromatogram of tryptophan obtained during the analysis of pineapple juice; Table S1: Some of the previously reported methods and samples analyzed for the determination of Trp; References of Table S1. References [39,40,41,42,43,44,45,46] are cited in the supplementary materials.
