# Supplementary material for: A New Electrochemical Method to Determine Tryptophan in Fruit Juices: Development and Validation
Source: Foods. 2022 Jul 20;11(14):2149. doi: 10.3390/foods11142149 (PMC9315539; doi:10.3390/foods11142149)
Supplement: Supplementary file 1 [file foods-11-02149-s001.zip › foods-1779734-supplementary.pdf]

foods MDPI

Supplementary Materials

A New Electrochemical Method to Determine Tryptophan in Fruit Juices: Development and Validation

Assefa Takele [1,2], José María Palacios-Santander [1,*] and Miguel Palma [2,*]

[1] Institute of Research on Electron Microscopy and Materials (IMEYMAT), Campus de Excelencia Internacional del Mar (CEIMAR), Department of Analytical Chemistry, Faculty of Sciences, University of Cadiz, Campus Universitario de Puerto Real, Polígono del Río San Pedro S/N, Puerto Real, 11510 Cádiz, Spain; asetak@yahoo.com

[2] Wine and Agrifood Research Institute (IVAGRO), Campus de Excelencia Internacional Agroalimentario (ceiA3), Department of Analytical Chemistry, Faculty of Sciences, University of Cadiz, Campus Universitario de Puerto Real, Polígono del Río San Pedro S/N, Puerto Real, 11510 Cádiz, Spain

* Correspondence: josem.palacios@uca.es (J.M.P.-S.); miguel.palma@uca.es (M.P.)

Table S1. Some of the previously reported methods and samples analyzed for the determination of Trp.

Method	Real Sample	Sample Preparation	LOD	LOQ	Reference
High performance liquid chromatography	Human serum and plasma samples	Deproteinized, vortexed, and centrifuged for 10 min at 15,000 g at 4 °C		3.97 µM	[2]
	Milk and sport supplements formulae	Hydrolyzed and centrifuged at 10,000 g for 4 min and filtered through 0.2 µm nylon filter membrane	0.46 µM	1.37 µM	[39]
	Infant formulae	Hydrolyzed by heating at 120 °C for 8 h and filtered through a membrane filter	0.005 µM	0.01 µM	[40]
	Yoghurts	Diluted in distilled water, centrifuged for 10 min at 3000 rpm, and ultrafiltered through an ultrafiltration cell	Not reported	Not reported	[41]
	Human hair	Alkaline hair hydrolysis, incubated and neutralized and filtered through a 0.45 µm Millipore® filter	0.012 µM	0.035 µM	[42]
	Cereals and legumes, such as wheat, rye, barley, chickpea, lentil, and kidney beans	Alkaline hydrolysis in an oven, cooled and filtered through ashless filter papers and 0.20 µm filter	0.009 µM	0.029 µM	[11]
Liquid chromatography coupled with mass spectrometry (LC–MS/MS)	Human plasma	Mixed with activated charcoal and shaken with orbital shaker, centrifuged at 15,000 g for 15 min, and filtered through 0.45 µm filter	0.083 µM	0.139 µM	[6]
Ultra performance liquid chromatography coupled with mass spectrometry (UPLC–MS/MS)	Fermented foods, such as beer, red wine, bread, yoghurt, and white cheese	Liquid samples were diluted and filtered through a 0.45 µm syringe filter; lyophilized samples vortexed and centrifuged at 3000 g for 5 min and filtered through a 0.45 µm syringe filter	0.007-0.181 µM	0.023-0.605 µM	[43]

	Mice tissue	Homogenized with a Precellys homogenizer and extracted with ice-cold MeOH (v/v)) and centrifuged	0.008 µM		[44]
Ultra performance liquid chromatography	Rice grain extracts	Extracted using ultrasound-assisted extraction (UAE)	7.44 µM	22.52 µM	[38]
Electroanalytical methods	Human serum and urine samples	Serum samples vortexed, centrifuged for 3 min at 6000 rpm, and filtered; urine samples centrifuged and filtered	0.03 µM	0.1 µM	[1]
	Glutathione tablet, hemolyzed erythrocytes, and urine samples	Not reported	Not reported	Not reported	[45]
	Amino acid preparations	Diluted using 0.1 M PBS (pH 7.0) and measured	0.31 µM	Not reported	[34]
	Pharmaceutical formulations (tablet)	Pulverized in a mortar and dissolved in the buffer and treated in an ultrasonic cleaner	1.7 µM	Not reported	[8]
	Blood serum, saliva, urine, and milk samples	Diluted with 0.01 M PBS	0.00036 µM	Not reported	[46]

LOD: limit of detection; LOQ: limit of quantitation; PBS: phosphate-buffered saline.

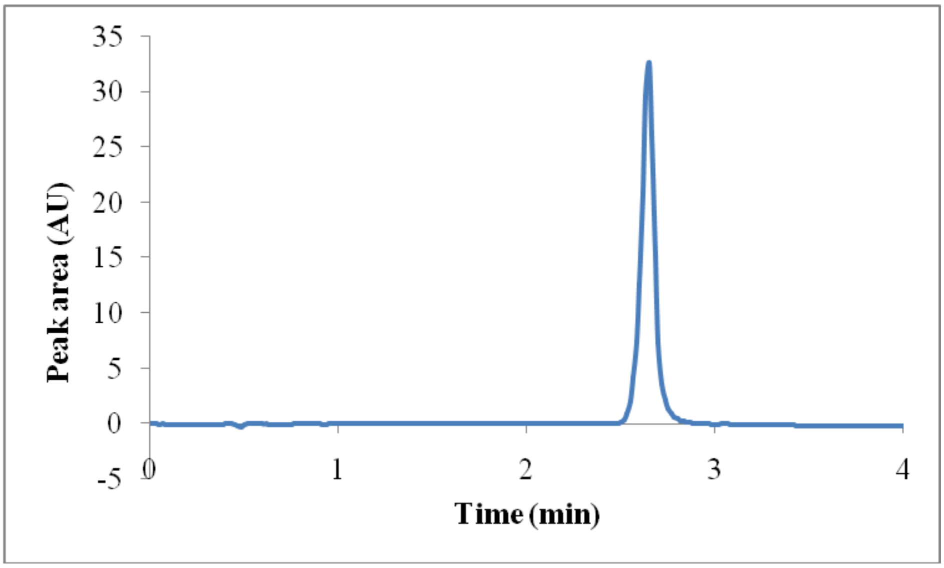

Figure S1. Chromatogram of tryptophan obtained during the calibration curve.

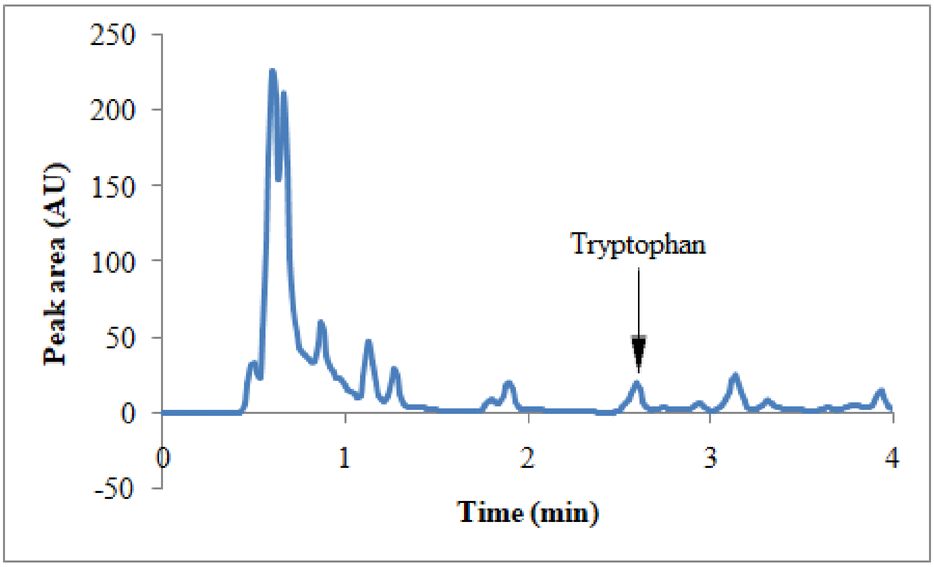

Figure S2. Chromatogram of tryptophan obtained during the analysis of peach + grape juice.
